# Diagnostic accuracy and radiological validation of intracerebral hemorrhage diagnosis in the Swedish Stroke Register (Riksstroke)

**DOI:** 10.1111/ene.16413

**Published:** 2024-07-15

**Authors:** Gabriella Sultani, Amir Hillal, Birgitta Ramgren, Trine Apostolaki‐Hansson, Bo Norrving, Johan Wasselius, Teresa Ullberg

**Affiliations:** ^1^ Department of Neurology Skåne University Hospital Malmö Sweden; ^2^ Department of Clinical Sciences Lund University Lund Sweden; ^3^ Medical Imaging and Physiology Skåne University Hospital Lund Sweden

**Keywords:** diagnostic accuracy, intracerebral hemorrhage, radiological validation, stroke register

## Abstract

**Background and purpose:**

National quality registries for stroke care operate under the assumption that the included patients are correctly diagnosed. We aimed to validate the clinical diagnosis of spontaneous intracerebral hemorrhage (ICH) in Riksstroke (RS) by evaluating radiological data from a large, unselected ICH population.

**Methods:**

We conducted a retrospective, multicenter study including all ICH patients registered in RS between 2016 and 2020 residing in Skåne County in Sweden (1.41 million inhabitants). Radiological data from first imaging were evaluated for the presence of spontaneous ICH. Other types of bleeds were registered if a spontaneous ICH was not identified on imaging. The radiological evaluation was independently performed by one radiology fellow and one senior neuroradiologist.

**Results:**

Between 2016 and 2020, 1784 ICH cases were registered in RS, of which 1655 (92.8%) had a radiological diagnosis consistent with spontaneous ICH. In the 129 (7.2%) remaining cases, the radiological diagnosis was instead traumatic bleed (*n* = 80), subarachnoid hemorrhage (*n* = 15), brain tumor bleed (*n* = 14), ischemic lesion with hemorrhagic transformation (*n* = 14), ischemic lesion (*n* = 3), or no bleed at all (*n* = 3). There was a higher degree of incorrect coding in the older age groups.

**Conclusion:**

At radiological evaluation, 92.8% of ICH diagnoses in RS were consistent with spontaneous ICH, yielding a high rate of agreement that strengthens the validity of the diagnostic accuracy in the register, justifying the use of high coverage quality register data for epidemiological purposes. The most common coding error was traumatic bleeds that were classified as spontaneous ICH.

## INTRODUCTION

Intracerebral hemorrhage (ICH) accounts for 13% of acute stroke cases in Sweden and up to 30% worldwide [1, 2]. The 90‐day mortality rate is approximately 40%, and only one in five patients regains functional independence [3]. Further research is needed to highlight existing inadequacies in the management of ICH and to identify strategies to address these areas of improvement.

National quality registries provide an excellent data source to monitor health care and evaluate the introduction and implementation of treatment protocols, provided that they have high coverage and accuracy [4]. Several examples exist where national register data have provided valuable information about the safety and efficacy of novel treatments regarding ischemic stroke [5, 6, 7]. Although new evidence‐based treatments have been scarce in regard to ICH, improved therapies are emerging [8]. To perform reliable evaluations of health care performance on a nationwide level, national quality registries must obtain high coverage, and key variables should be independently validated to ensure the internal and external validity of data [9]. Riksstroke (RS), the Swedish national quality register for stroke care, has high coverage (>90%) of all hospital admissions for stroke and has been extensively used for research purposes [10], but the accuracy of ICH diagnoses has yet to be independently validated.

The aim of this study was to assess the accuracy of registered ICH diagnoses in RS compared to radiological evaluation as gold standard and to classify wrongly coded patients into diagnostic categories.

## METHODS

### Study design

We conducted a diagnostic accuracy study based on data from RS where the coding of ICH in the register was validated against the radiological diagnosis. The article follows the standards of reporting in accordance with STARD (Standards for Reporting Diagnostic Accuracy Studies) 2015 [11]. We studied ICH patients residing in Skåne County (1.4 million inhabitants) [12], including one comprehensive and nine primary stroke centers.

### Participants

Patients were included if they were aged ≥18 years, residing in Skåne County, and registered with an ICH diagnosis (International Classification of Diseases, 10th Revision [ICD‐10] I61) in RS during 2016–2020. Patients were excluded if their initial head computed tomography (CT) scan was unavailable in the picture archiving and communication system (PACS) or if the CT scan was not possible to evaluate due to substantial technical artifacts.

Spontaneous ICH was defined as ICH caused by deep perforating vasculopathy, cerebral amyloid angiopathy (CAA), vascular malformations, intracranial venous thrombosis, brain surgery, or other rare conditions [13]. Intracerebral hemorrhage due to an underlying brain tumor or metastases, as well as hemorrhagic transformation of an ischemic lesion, is by definition spontaneous ICH. However, conforming to the stroke registration guidance manual for RS [14], they were not included in this study. Hemorrhagic transformation of an ischemic lesion is not registered as ICH in RS, as the primary diagnosis is considered to be ischemic stroke [14]. For this reason, these two conditions will not be included when referring to spontaneous ICH henceforth in this study. Furthermore, by definition, spontaneous aneurysmatic/nonaneurysmatic subarachnoid hemorrhage (SAH) is not defined as an ICH and is therefore not included in this group. In cases of nonaneurysmatic SAH, CAA may have been an underlying pathology, although this was not further analyzed in patients without accompanying ICH identified on imaging. Traumatic bleeds were defined as isolated or combinations of SAH, epidural hematoma, subdural hematoma, or ICH, with a history of head trauma, presence of neck/trauma on CT, or presence of a skull fracture. Cerebral contusions were defined as punctate/large hyperdense foci/hematomas involving the grey matter and subcortical white matter. Usual locations of cerebral contusions are the anterior cranial fossa, inferior frontal lobes, and anterior–inferior temporal lobes, in addition to other locations depending on the direction of the head strike [15]. Traumatic SAH was defined as sulcal SAH occurring at the site of impact (coup) or opposite the site of impact (contrecoup).

Cases where traumatic or spontaneous ICH could not be distinguished were classified as spontaneous ICH (*n* = 15).

### Baseline data variables

To ensure external validity, aggregated data from the entire Swedish stroke population in RS were compared with the baseline and clinical characteristics of patients residing in Skåne County. All patient characteristics were collected from RS except for oral anticoagulation treatment at the time of ICH diagnosis, which was collected from the Swedish Prescribed Drugs Register.

### Data sources

#### Riksstroke

RS is the Swedish quality register for stroke care. All Swedish hospitals (*n* = 72) admitting patients with stroke contribute to data collection, resulting in high coverage (>90%) of Swedish hospital‐admitted stroke cases. The setting in which the ICD‐10 classification was applied in this study was if the patient included in the registry was treated in a hospital setting or had sought health care [10]. The register contains data on patient characteristics, provided care, and patient outcomes. The data are collected by licensed health care workers through the completion of RS's acute phase questionnaire. All patients registered in RS are informed of registration and handling of their patient data for research purposes. Approximately 2600 ICH cases are annually registered in RS [1].

#### Radiological database

Skåne County has a common PACS where radiological data from all hospitals in the region are gathered. By using the PACS system, access was granted to all available imaging data from hospitals providing stroke care within the region.

### Radiological evaluation

Radiological data from the initial head CT scan following ICH were evaluated to verify the presence of a spontaneous ICH. If not present, other types of bleeds were categorized. The radiological evaluation was performed by one radiology fellow with 1 year of neuroradiology experience who examined and evaluated the CT scans for all included patients (*n* = 1794). For interrater agreement assessment, the initial 500 scans were also independently evaluated by a senior neuroradiologist with >20 years of experience. Prior to the image evaluation, both raters made consensus reading of >50 other ICH cases to ensure an even standard of assessment. Any disagreements were resolved by consensus. To distinguish between spontaneous ICH and other types of bleeds, the assessors looked for typical radiological patterns and signs of causes other than spontaneous ICH [16]. Other available imaging (e.g., CT angiography [CTA], magnetic resonance imaging [MRI], or magnetic resonance angiography [MRA]) and the radiological referral text in PACS were also used for the radiological evaluation of ICH when required. Medical records, other than the radiological referral text in PACS, were not reviewed in this study.

### Statistical analysis

IBM SPSS statistics version 28 was used for all statistical analyses. Cohen kappa was used to assess interrater agreement for the presence of spontaneous ICH. The data were analyzed using standard descriptive statistics. After reviewing the radiological data, the ICH diagnosis registered in RS was defined as true positive (TP) if it was consistent with the radiological evaluation of ICH, or false positive (FP) if this was not the case. The positive predictive value (PPV = TP / [TP + FP]) was expressed as a percentage. We calculated the PPV for the total study population and stratified patients into the following age categories: <65 years, 65–74 years, 75–84 years, and ≥85 years. The proportions of patients incorrectly coded in RS were compared between the different age groups using chi‐squared test. Probability values (two‐sided) < 0.05 were considered statistically significant.

## RESULTS

### Participants

The inclusion criteria were fulfilled in 1794 patients. In total, 10 patients were subsequently excluded from the study due either to missing the initial head CT (*n* = 8) or to the presence of substantial image artifacts (*n* = 2), resulting in a final study population of 1784 patients.

Baseline and clinical characteristics for the study population and the entire Swedish ICH population between 2016 and 2020 are shown in Table 1. Median age for both groups was 76 years, and male sex represented approximately 55% of the total patient population. Most clinical parameters were similar between the two groups, except for admission to a stroke unit, which was 62% in Skåne County compared to 56% in the entire Swedish ICH population, and admission to an intensive care unit, which was correspondingly 13% and 21%.

**TABLE 1:** Baseline characteristics of patients with ICH in the Skåne County population and in the entire Swedish ICH population during the study period (2016–2020).

Baseline characteristics	Skåne County population, *n* = 1786, *n* (%)	Swedish population, *n* = 13,980, *n* (%)
Age, years, median (IQR)	76 (67–84)	76 (66–83)
Gender, male	1002 (56.1)	7724 (55.3)
Hypertension	1058 (59.2)	8190 (58.6)
Diabetes	334 (18.7)	2422 (17.3)
Previous stroke	439 (24.6)	3158 (22.6)
Level of consciousness		
Alert	986 (55.2)	8446 (60.4)
Drowsy	448 (25.1)	3135 (22.4)
Comatose	336 (18.8)	2399 (17.2)
OAC at onset	398 (22.3)	2943 (21.1)
Admitted to regular care unit	185 (10.4)	1717 (12.3)
Admitted to stroke unit	1109 (62.1)	7825 (56.0)
Admitted to intensive care unit	239 (13.4)	2960 (21.2)
Admitted to neurosurgical ward	45 (2.5)	412 (2.9)

*Note*: The proportion of missing data varied between 0% and 1.1% for all variables.

Abbreviations: ICH, intracerebral hemorrhage; IQR, interquartile range; OAC, oral anticoagulation.

### Radiological evaluation

**FIGURE 1:**
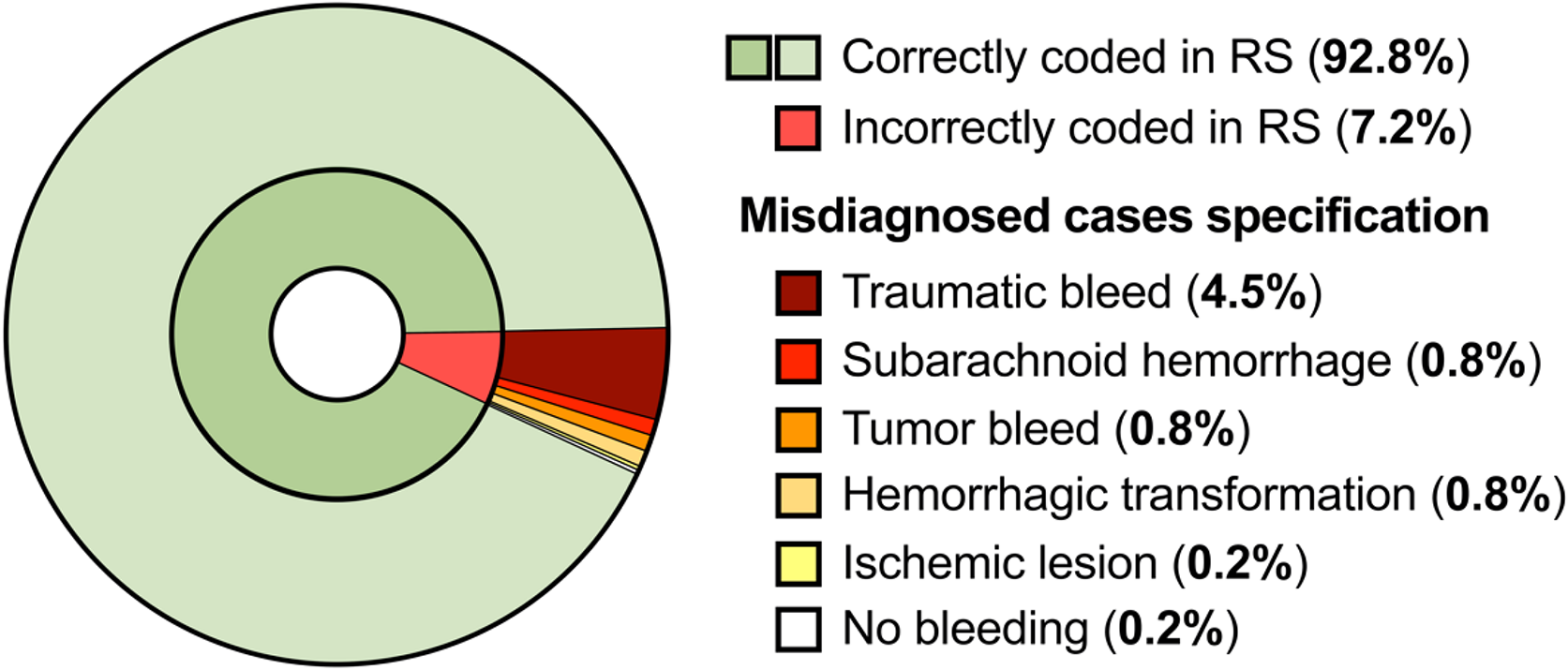
Donut chart demonstrating the proportions of patients incorrectly coded in Riksstroke (RS), as well as a specification of the different diagnoses and their proportions in this group.

In total, 1784 ICH cases were evaluated, of which 1655 cases were radiologically consistent with the diagnosis of spontaneous ICH. In all cases, first imaging was conducted using CT scans; none of the initial scans was performed using MRI. This resulted in a PPV of 92.8%. For the remaining 129 cases (7.2%), the radiological assessment was consistent with traumatic bleed (*n* = 80), SAH (*n* = 15), tumor bleed (*n* = 14), ischemic lesion with hemorrhagic transformation (*n* = 14), ischemic lesion (*n* = 3), or no bleed at all (*n* = 3; Figure 1). Disagreement between the two independent raters occurred in four of the 500 cases, yielding an interrater agreement of 99.2% (kappa = 0.96). All cases of disagreement were subsequently resolved by consensus.

**FIGURE 2:**
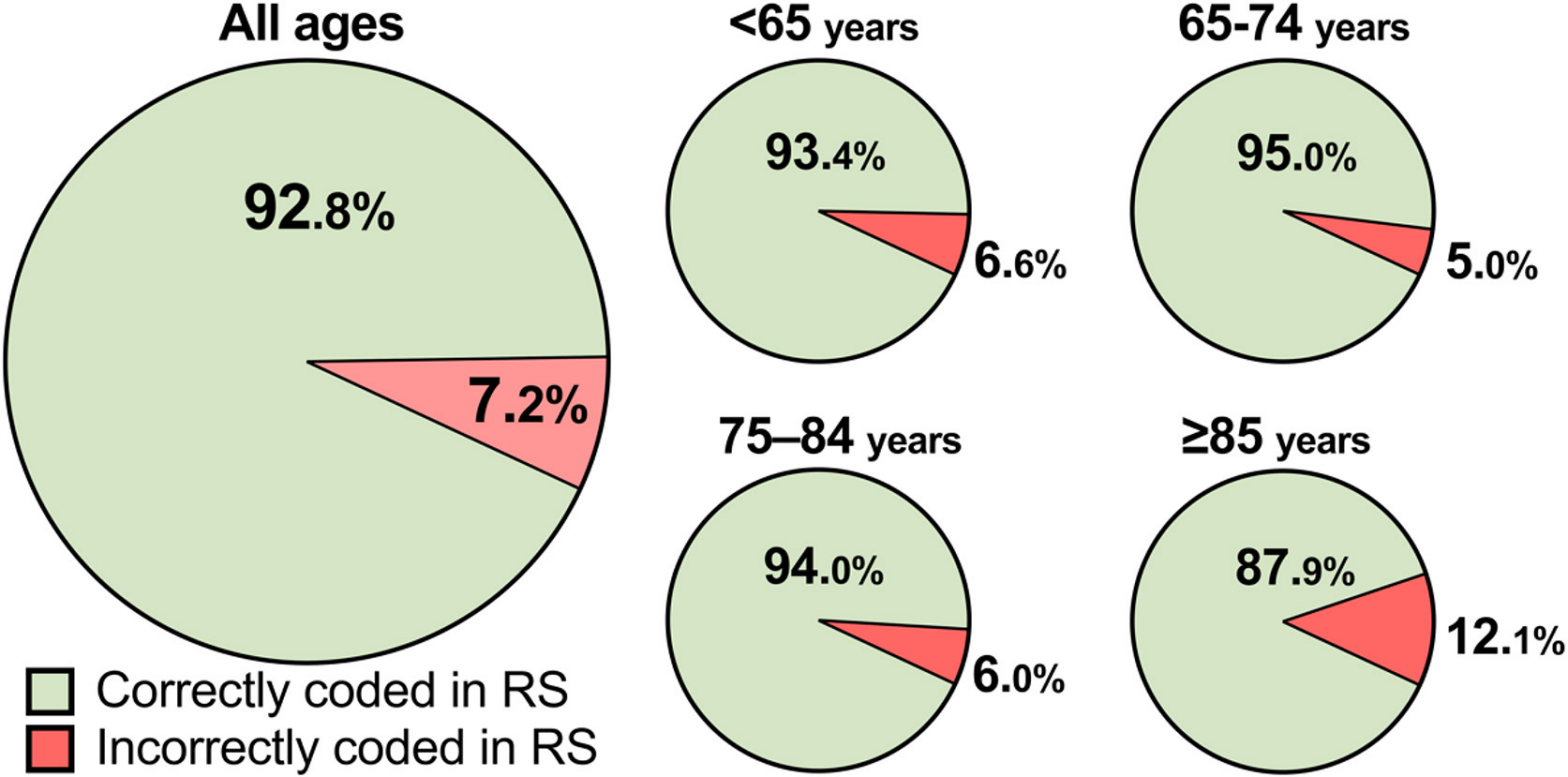
Pie charts demonstrating the proportions of incorrectly coded cases in Riksstroke (RS) in the entire study population as well as for the four different age categories.

Diagnoses after radiological evaluation for the total study population, in addition to diagnosis stratification by age, are shown in Table 2. The proportion of patients incorrectly coded in RS as having a spontaneous ICH was 12.1% in patients aged ≥85 years compared to 5.0%–6.6% in younger age groups (*p* < 0.001; Figure 2). Specifically, traumatic bleeds that were incorrectly diagnosed as spontaneous ICH were more prevalent in patients aged ≥85 years. Regarding tumor bleeds, the tumor was known and MRI‐verified prior to the ICH diagnosis in 13 of the 14 cases that were wrongly classified as spontaneous ICH in RS.

**TABLE 2:** Table of diagnoses upon radiological evaluation in the total study population and in different age groups within the study population.

Diagnosis	All ages, *n* = 1784, *n* (%)	<65 years, *n* = 378, *n* (%)	65–74 years, *n* = 424, *n* (%)	75–84 years, *n* = 584, *n* (%)	≥85 years, *n* = 398, *n* (%)
Spontaneous ICH	1655 (92.8)	353 (93.4)	403 (95.0)	549 (94.0)	350 (87.9)
Traumatic bleed	80 (4.5)	8 (2.1)	12 (2.8)	24 (4.1)	36 (9.0)
Subarachnoid hemorrhage	15 (0.8)	7 (1.9)	1 (0.2)	2 (0.3)	5 (1.3)
Tumor bleed	14 (0.8)	5 (1.3)	4 (0.9)	3 (0.5)	2 (0.5)
Ischemic lesion with hemorrhagic transformation	14 (0.8)	3 (0.8)	3 (0.7)	5 (0.9)	3 (0.8)
Ischemic lesion	3 (0.2)	1 (0.3)	1 (0.2)	0 (0.0)	1 (0.3)
No bleeding	3 (0.2)	1 (0.3)	0 (0.0)	1 (0.2)	1 (0.3)

Abbreviation: ICH, intracerebral hemorrhage.

## DISCUSSION

In our study population of 1784 individuals, the assessment of radiological data was consistent with RSs diagnosis of spontaneous ICH in 92.8% of cases, yielding a PPV of 93%.

Previous studies on the validation of ICH diagnoses using administrative or register data have reported PPVs ranging from 66% to 97% [17, 18, 19, 20, 21, 22], although these studies were largely based on the review of medical records and not on the assessment of radiological images. Several of these studies were not solely focused on ICH, but on the validation of stroke diagnoses in general, including ischemic stroke and/or SAH [18, 19, 20, 21, 22]. In several of the studies [17, 18, 20, 21, 22], the number of ICH cases included was less than in ours. The study conducted by Gaist et al. was the only abovementioned study that included a comparable number of ICH cases, yet they reported a PPV of 73% for the diagnosis of ICH in the Health Improvement Network, a health care database from the United Kingdom [19]. A Norwegian study conducted by Oie et al. reported a PPV of 96.9% for the primary diagnosis of ICH in inpatient admissions registered in the Norwegian Patient Register (NPR) using information provided by electronic medical records (diagnostic imaging, radiology reports, and laboratory tests) [17]. As only radiology reports were screened, the determination of the consistency of the diagnosis of ICH in the NPR with the assessment of radiological data was not performed. Thus, certain patients may have been falsely coded due to misdiagnosis.

In our population, we identified that traumatic bleeds constituted more than half of all misdiagnosed cases and that faulty coding was more common in the elderly (≥85 years old). The finding that older patients have a higher rate of traumatic bleeds misdiagnosed/miscoded as spontaneous ICH may potentially be explained by the higher prevalence of traumatic bleeds and intracranial hematomas in older age groups. Another possible explanation is the finding that elderly stroke patients more often receive care outside neurological wards [23], where physicians may be less accustomed to diagnostic coding of ICH. Traumatic bleeds may be incorrectly coded as spontaneous ICH in RS, because the ICD‐10 code for traumatic ICH (S06.3) reads “focal brain injury” and not “traumatic ICH.” This could presumably pose a challenge for physicians to identify the correct diagnostic code, and they may instead apply the code for spontaneous unspecified ICH (I61.9), a code that should only be used in cases of spontaneous ICH [24]. With the additional training of health care personnel in diagnostic coding, there is potential to improve the diagnostic accuracy of stroke, and thus further improve the validity of the quality register.

Differentiating between traumatic versus nontraumatic ICH was ambiguous in 15 patients in whom a history of head trauma was reported but CT revealed only an isolated ICH and no imaging findings of traumatic injuries. In these instances, patients were diagnosed with spontaneous ICH. It was unclear and indistinguishable based on the CT findings alone whether these patients first had an ICH followed by a traumatic fall or vice versa. Furthermore, tumor bleeds constituted only a small minority of cases (<1%). Although a small number overall, the proportion of previously known tumors in patients with tumor bleeds was high (13/14 cases). Because the stroke registration guidance manual for RS clearly states that tumor bleeds should not be registered in RS, we expected the number of previously known tumors to be smaller.

The number of patients diagnosed with ICH due to vascular malformations, such as arteriovenous malformations or cavernomas, was lower than expected. One potential reason for this is that only a minority of patients in our study population underwent radiological examination with CTA and/or MRI/MRA. The increased use of these imaging modalities in the diagnostic workup of ICH patients would improve the causal classification of ICH. The causal classification of ICH is of importance because the management, recurrence risk, and prognosis may differ based on the underlying etiology [25]. Because CTA and/or MRI are not extensively used in the diagnostic workup of ICH in Sweden, the underlying etiology of spontaneous ICH may be unknown in a number of patients included in this study. Due to the potential absence of an adequately comprehensive diagnostic workup to identify an underlying cause of ICH, there is a small possibility that misclassification of spontaneous ICH may have occurred if, for example, an underlying tumor was not initially identified on CT. Nonetheless, we presume that this number is uninfluential to our results, because patients are to be included in RS regardless of the exact underlying cause of spontaneous ICH (except for tumor bleeds and the hemorrhagic transformation of an ischemic lesion). The annual RS register report has shown an upward trend when it comes to diagnostic workup with MRI and CTA in ICH patients. According to the annual report from 2020, the proportion of ICH patients examined with CTA and/or MRI was 32% and 16%, respectively. The proportion of patients undergoing these examinations was higher in the younger population, correspondingly 55% and 37% in patients <55 years old [26]. Compared to other national cohorts, the use of MRI following ICH during the acute phase is low in Sweden. In a patient cohort of 3572 patients registered in the Swiss Stroke Register, approximately 40% of patients underwent MRI within 90 days of ICH [27]. RS does not include data on MRI investigations performed after patient discharge. Therefore, MRI use in Sweden is probably underestimated in this study.

Establishing national‐ and regional‐level systems for assessing stroke clinical services, providing peer support for quality improvement, and making audit data routinely available to the general public are among the goals of the Stroke Action Plan for Europe [28]. However, national quality registers are currently available in fewer than half of the European countries [29]. To provide reliable data, it is essential that definitions and terminology are standardized and that data for main stroke subtypes are validated separately.

### Strengths

The strengths of this study include the large sample size based on an unselected patient cohort. The patient cohort represents a large Swedish region, encompassing approximately 13% of the total Swedish population, which contributes to the generalizability of this study [30]. Second, the number of missing CT examinations was low, and the coverage of stroke cases in RS during the study period was high. The study covers 10 hospitals ranging in size from university hospitals to county and local hospitals. Therefore, in addition to the large number of patients, the study population can be considered representative of the entire Swedish ICH population, which is also seen in Table 1, as the baseline and clinical characteristics of the two populations are similar.

### Limitations

As this study validated the diagnosis of ICH registered in the Swedish Stroke Register, RS, results from this study may not be generalizable to other stroke registers. Selection bias is a second limitation of our study, because any ICH patient not included in RS is also missed in our analysis. In a recent study on case ascertainment in Swedish hospital‐based stroke registries, including RS and the Lund Stroke Register, by Aked et al. [31], it was demonstrated that, despite best efforts, there would always be stroke cases that are missed. Fifteen percent of the patient population in the aforementioned study had ICH; the remaining had ischemic stroke (84%) or underdetermined stroke cause (1%). In the small ICH population included in this study, case ascertainment in RS was 83% (*n* = 50/60). In the total patient population, patients who were not identified by RS more often had less severe strokes or a higher early case fatality. As the ICH population in this study was small, the generalizability of these results to the entire country is low. Thus, even though the number of missing ICH cases in RS is estimated to be low given the high coverage of RS [1], it is not possible to estimate the number of false negative cases, that is, patients with ICH who were not registered in RS, in this study.

## CONCLUSIONS

In conclusion, our study shows a high rate of agreement between ICH diagnoses registered in RS and the radiological evaluation. Our results strengthen the validity of the diagnosis of ICH in RS and support the use of the register for research and quality evaluation. Due to its high coverage and high validity, RS is a register that can be appropriately used for epidemiological studies. Although our results confirm a high diagnostic accuracy, they also identify areas for improvement, such as the coding of traumatic bleeds and bleeds in patients with underlying brain tumors. The current trend seen in the increased use of CTA and/or MRI may further improve the causal classification of ICH.

## AUTHOR CONTRIBUTIONS

**Gabriella Sultani:** Conceptualization; writing – original draft; formal analysis; writing – review and editing. **Amir Hillal:** Data curation. **Birgitta Ramgren:** Data curation; writing – review and editing. **Trine Apostolaki‐Hansson:** Writing – review and editing; supervision. **Bo Norrving:** Conceptualization; supervision; writing – review and editing. **Johan Wasselius:** Conceptualization; supervision; writing – review and editing. **Teresa Ullberg:** Conceptualization; supervision; writing – review and editing.

## FUNDING INFORMATION

This study was funded by grants from the Crafoord Foundation (20200548), VINNOVA (2021‐02617), SUS Stiftelser & Fonder, Södra Sjukvårdsregionens doktorandanslag, and ALF Region Skåne.

## CONFLICT OF INTEREST STATEMENT

J.W. is a founder and shareholder in Uman Sense. T.U. has received honoraria for an expert group assignment from AstraZeneca. B.N. has received honoraria for serving on a data and safety monitoring board for the HOVID trial (Simbec‐Orion). The other authors have no conflicts of interest to disclose.

## ETHICS STATEMENT

The Swedish Ethical Review Authority approved the study (#2020–06800) and waived individual informed consent.

## Data Availability

An anonymized dataset supporting the conclusions of this article may be provided upon reasonable request.
